# Molecular cloning and characterization of Izumo1 gene from bovine testis

**DOI:** 10.1186/s40781-015-0049-1

**Published:** 2015-04-09

**Authors:** Ekyune Kim

**Affiliations:** College of Pharmacy, Catholic University of Daegu, Gyeongsan, 712-702 Republic of Korea

**Keywords:** Fertilization, Immunoglobulin, Sperm specific protein, IgSF family

## Abstract

A well-characterized sperm specific protein of the Member of immunoglobulin superfamily, IZUMO1, has crucial role in fertilization by mediating sperm binding to the egg plasma membrane in the mouse. However little is known about IZUMO1 in bovine. Here, we describe the molecular cloning and expression analysis of bovine IZUMO1 (bIZUMO1). RT-PCR and Western blot analysis of the bovine tissues indicated that bIZUMO1 was specifically expressed in the testis and sperm, Furthermore, the result of our biotinylation assay from ejaculated bovine sperm strongly suggest the assumption that bIZUMO1 is localized on the cell surface. These data imply the potential role of bovine IZUMO1 in mammalian fertilization.

## Background

The germ specific IgSF (Member of immunoglobulin superfamily) family of glycoprotein comprises one of the largest families of male infertility factors essential for spermatogenesis and sperm-egg interaction. It tightly regulates several aspects of biological processes, including degradation of the cumulus mass, sperm-zona pellucida (ZP) of egg and fusion of the egg plasma membrane after penetration of sperm into zona pullucida [1-3]. Intriguingly, many infertility cases exist anti sperm antibodies in either the male or female partner of an infertile couple, as cause of dysfunctions above mentioned mechanisms [4]. In 2005, the Okabe research group identified the mouse IZUMO1 protein, originally known as OBF13, as a novel member of the immunoglobulin superfamily (IgSF), containing a single immunoglobulin (Ig) domain in the extracellular domain. They found that it is expressed on the inner acrosomal membrane of mature sperm, suggesting that the Ig domain is exposed on the sperm surface after the acrosome reaction [5-7]. Although mouse IZUMO1 is absolutely essential for gamete fusion, its expression pattern in different species remains unknown [8]. In particular, research on bovine reproduction could provide a concrete basis on the molecular biology underlying animal fertilization, and thereby contribute to the understanding of specific gene functions related to human reproduction.

As IZUMO1 is critical for sperm–egg fusion in mice, understanding its expression and function in different animals may be of great relevance. IZUMO1 is encoded by a single-copy gene on mouse chromosome 7, but until now, IZUMO1 had not yet been identified in bovine. In the present study, we cloned and characterized the bovine IZUMO1 (bIZUMO1) gene and subsequently, generated a specific polyclonal antibody against IZUMO1 to identify its expression pattern and subcellular localization.

## Methods

### Animals

Fresh bovine tissues were purchased from local slaughterhouse (Daegu, Korea). The animal tissues in this study were used under the Guidelines of the Institutional Animal Care and Use Committee of Catholic University of Daegu.

### Total RNA extraction and reverse transcriptase polymerase chain reaction (RT-PCR)

Total RNA was obtained from bovine tissues using the Isogen kit (Nippon Gene, Toyama, Japan). Total RNA (5 μg) was reverse transcribed to cDNA with a SuperScript III First-Strand Synthesis System (Invitrogen, Carlsbad, USA). PCR amplification was carried out using EX Taq DNA polymerase (Takara, Ohtsu, Japan), according to the manufacturer’s instructions. One-tenth of the first-strand cDNA reaction mixture was used as a template for PCR. Specific PCR primers were designed for the amplification of the IZUMO1 genes from bovine. (GenBank Accession numbers: XM_002695199 respectively). Oligonucleotide sequences of all primers used in this study are as shown in Table 1. PCR was performed for 35 cycles of 94°C for 60 sec, 60°C for 60 sec, and 72°C for 60 sec in a PCR thermal cycler (Biometra Göttingen, Germany).

**Table 1:** **Primer sequences for characterization of bovine IZUMO1**

	**Sense primer (5′ to 3′)**	**Anti sense primer (5′ to 3′)**
RT-PCR	GAGCGCGAAGTCAAGGTCCATC	GCCGTCTCATCCTGAGTTTCGGTGT
Anti-body	CTCGAGGTTGTCGATGAGGCCACACTG	TCTAGAGTCCAGGATCATGTCTTCCAT

The PCR products obtained from the testis tissue of bovine was cloned and sequenced. In brief, after electrophoresis in a 1% agarose gel, the desired PCR bands were excised with a razor blade. Gel fragments were purified using a gel extraction kit (Qiagen, USA) according to manufacturer’s guidelines. Purified DNA fragments were cloned into pGEM-T-easy, and propagated in *Escherichia coli* DH5α. After isolation of plasmid DNA, inserts were sequenced with vector-specific T7 and SP6 sequencing primers (Promega, USA) on a 3730XL DNA analyzer (Applied Biosystems, USA).

### Antibodies

DNA fragment encoding the residues 40–137 of the bIZUMO1 protein was amplified by PCR, introduced into the pCold Vector (Takara, Japan), and expressed in *E. coli* BL21 (DE3) (Table 2). The recombinant His-tagged proteins were emulsified with Freund’s complete adjuvant (Sigma-Aldrich) and injected intradermally into female New Zealand white rabbits [9]. After fractionation of the antisera with ammonium sulfate (0–40% saturation), anti-bIZUMO1 antibody was affinity-purified on a Melon Gel IgG purification resin (Thermo Scintific, Rockford, IL USA).

**Table 2:** **IZUMO1 amino acid sequence homology among bovine, mouse and human**

	**Ig domain (%)**
bIZUMO1/hIZUMO1	79.8
bIZUMO1/mIZUMO1	64.1
bIZUMO1/pIZUMO1	86.6

### Preparation of protein extracts

Various bovine tissues were chilled on ice for 2 h and subjected to a lysis buffer consisting of 20 mM Tris–HCl, pH 7.4, 1% Triton X-100 (TX-100), 150 mM NaCl, and 1% protease inhibitor cocktail (Sigma-Aldrich) for the extraction of proteins [10]. After centrifugation at 10,000 g for 10 min at 4°C, proteins retained in the supernatant were analyzed.

### Western blot analysis

Denatured proteins were separated by sodium dodecyl sulfate-polyacrylamide gel electrophoresis (SDS-PAGE) and transferred onto Immobilon-P membranes (Millipore, USA). The blots were blocked with 2% skim milk followed by incubation with primary antibodies for 2 h and subsequently, with horseradish peroxidase-conjugated secondary antibodies for 1 h. Then, the immunoreactive proteins were detected using an ECL western blotting detection kit (Amersham Biosciences, Little Chalfnot, UK).

### Construction of expression vector and transfection into HEK293 cells

An expression vector of bIZUMO1 was constructed in pEGFP N1 vector (Clontech, Mountain View, USA). The primers 5′-CTCGAGGCCACCATGGATTATCTGCCTGGCCACCT-3′ and 5′-GGATCCAGCAGCTCGACTGCCAGAGCTGAAC-3′ were used to amplify the entire bIZUMO1 ORF from bovine testis cDNA. The amplified DNA was then digested with *Xho*I and *Bam*HI and sub-cloned into the pEGFP N1 vector. After we confirmed the integrity of the reading frame and cloning sites of the expression vector by DNA sequencing, the plasmid vector was transfected into HEK293 cells [11]. Briefly, HEK293 cells were cultured in Dulbecco’s modified Eagle medium supplemented with 10% fetal bovine serum. The cells were transiently transfected with the bIZUMO1 expression vector using ViaFect (Promega) according to the manufacturer’s protocols (Promega). Forty-eight hours after transfection, the transfected cells were washed 3 times in phosphate-buffered saline (PBS) and lysed in 1% Triton-100 (TX-100) supplemented with 1% protease inhibitor cocktail (Sigma-Aldrich). Western blotting was performed using 1:300 dilutions of anti-bIZUMO1 antibody, followed by incubation with a 1:3000 dilution of horseradish peroxidase-labeled goat anti-rabbit IgG. ECL detection of bands was performed as described in the previous section.

### Biotinylation of bovine sperm surface

Biotinylation of bovine sperm (2.5 × 10^7^/ml) were kept at room temperature for 1 h in PBS containing 1 mM sulfo-NHS-LC biotin (Pierce). The biotinylated sperm samples were washed twice with PBS and lysed with the above protein lysis buffer. Proteins were subjected to SDS-PAGE under reducing conditions followed by Western blot analysis [12].

## Results and discussion

### Isolation and characterization of bovine IZUMO1 (bIZUMO1)

**Figure 1:**
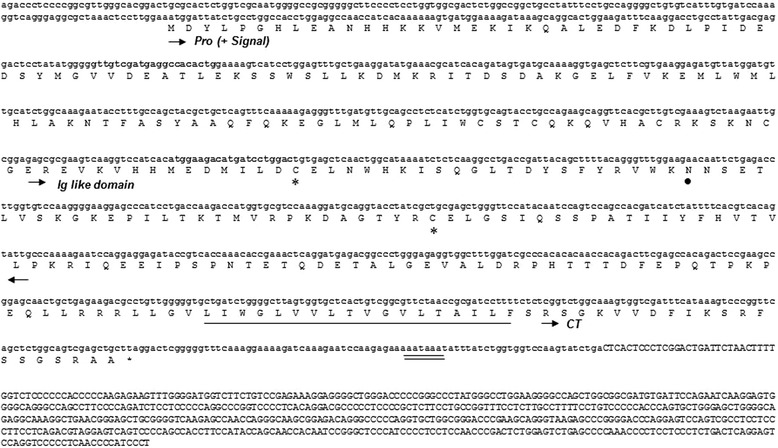
**Nucleotide and deduced amino acid sequence of bovine IZUMO1.** The deduced amino acid sequence is shown below the nucleotide sequence numbered in the 5′ to 3′ direction. Arrows indicate predicted boundaries of Immunoglobulin (Ig) like domain. The cysteine residues that might form a disulphide bridge are indicated by asterisks. A putative polyadenylation signal, AATAAA, is indicated by double underlines. The region of a putative transmembrane domain is underlined with solid line. CT, cytoplasmic tail.

**Figure 2:**
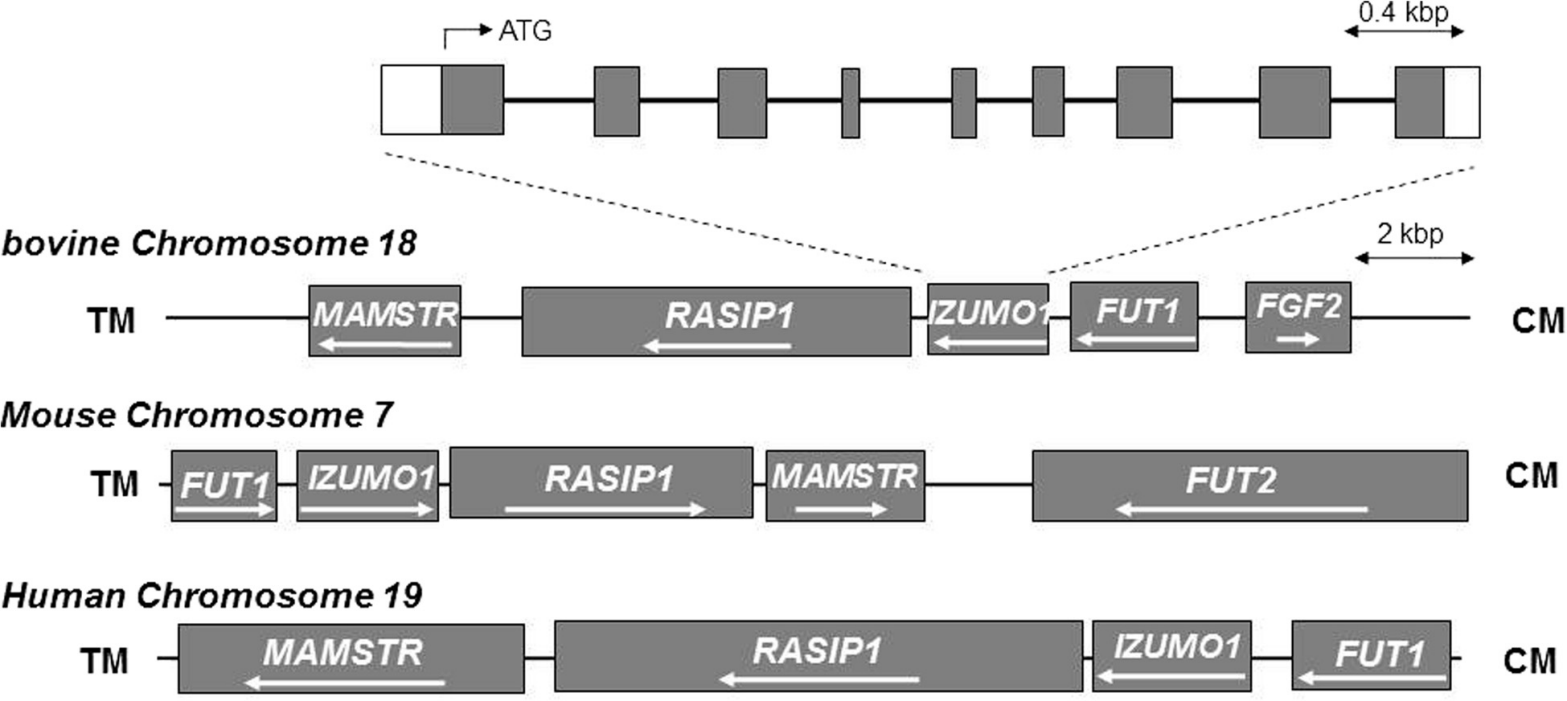
**Genomic structure of bovine IZUMO1 (bIZUMO1) on bovine chromosome 18.** The bIZUMO1 gene contains 9 exons (shaded boxes) interrupted by 8 introns. The translation initiation codon, ATG, is located in the first exon.

Since IZUMO1 is critical for sperm-egg fusion in mice, it is important to understand its expression and function in different animals. IZUMO1 is a single-copy gene in mouse chromosome 7, but bovine IZUMO1 (bIZUMO1) had not yet been identified. To determine if a bIZUMO1 gene exists, we initially searched the GenBank database derived from bovine testis. The National Center for Biotechnology Information (NCBI) database provides variant bIZUMO1 ORF. We used 3′ and 5′ rapid amplification of cDNA (RACE) to clone the missing sequence of the bIZUMO1 gene (Figure 1). Searches in the National Center for Biotechnology Information (NCBI) database (www.ncbi.nlm.nih.gov/genomes) indicated that the genomic bIZUMO1 sequence is located on porcine chromosome 18, containing 9 exons and 8 introns (Figure 2).

**Figure 3:**
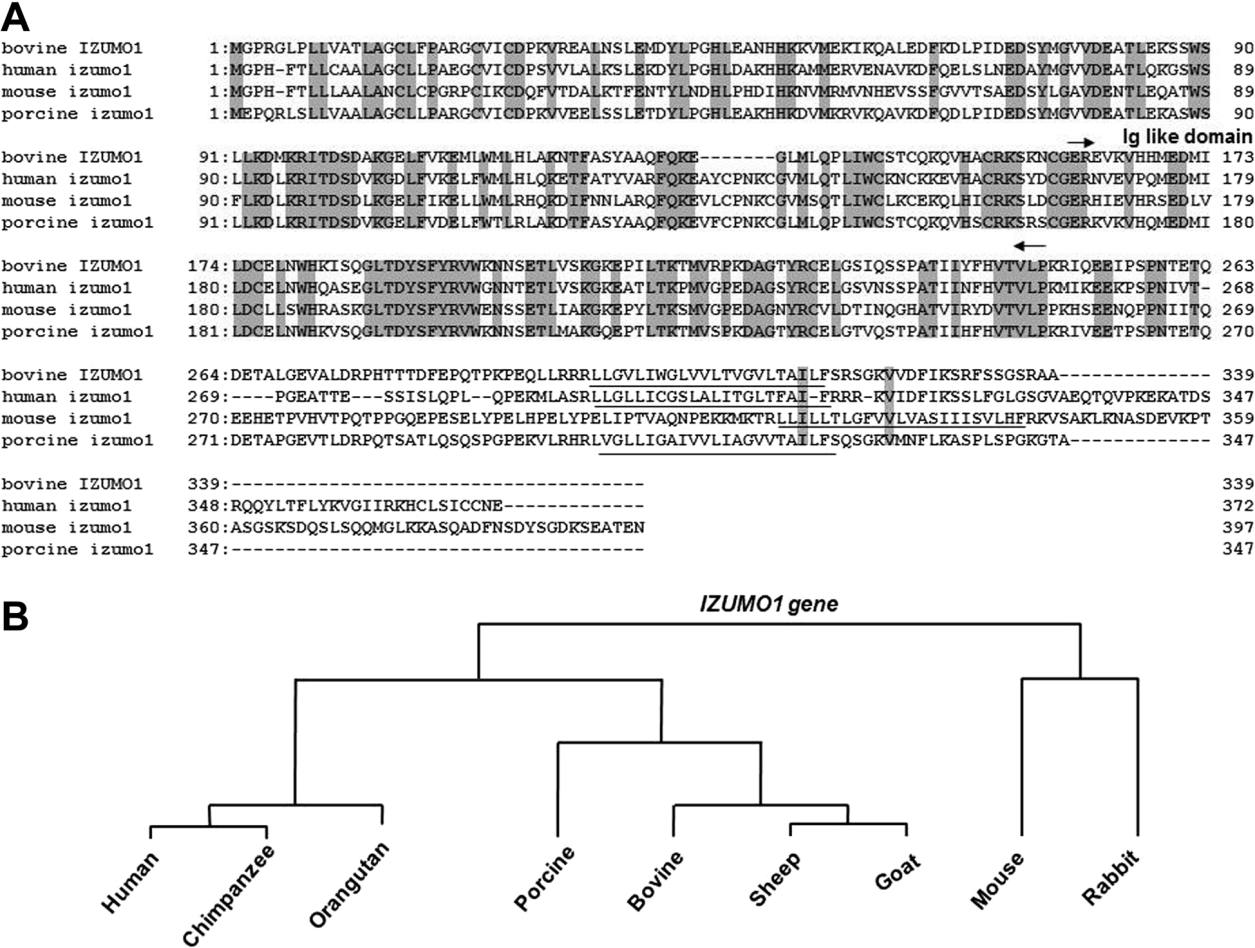
**Alignment of amino acid sequences of IZUMO1 homologues from various mammalian species. (A)** Amino acids identical in IZUMO1 proteins of bovine, human, mouse and pig. Amino-acid identity is represented by shaded boxes. Arrows indicate putative starting points of the signal sequence and the immunoglobulin (Ig) domain. The transmembrane domain is underlined. **(B)** Phylogenetic tree showing the similarity of amino acid sequences across mouse, rabbit, goat, sheep, cow, pig, chimpanzee, orangutan, and human IZUMO1 protein. These proteins have been highly conserved.

The DNA sequence indicated that bIZUMO1 is synthesized as a single-chain protein with a calculated molecular mass of 38,290 Da and comprising 339 amino acid residues. It is predicted to contain multiple domains, including a signal peptide, an immunoglobulin (Ig)-like domain, a transmembrane domain, and a cytoplasmic tail consisting (Figures 1A and 3).

The sequences of the bovine IZUMO1 showed 55.1% and 64.0% similarity to the mouse and human, respectively. Although the sequences of the N-terminus were highly conserved across species, the C-terminus sequences were divergent; there was very little homology between the sequences of the cytoplasmic tail domains (Figure 3 and Table 2). We found that there are three types of C-terminus regions in various IZUMO1 homologs: those with a larger C-terminal region, such as in the mouse and human; those with a somewhat shorter C-terminal, such as in humans; and those where the C-terminal consists of approximately 20 amino acid, such as in the bovine and pig (Figure 3). Thus, one of the main phylogenetic groups of IZUMO1 across species is the group where a single amino acid residue occupies the cytoplasmic tail region.

Given that the extracellular domains were highly conserved for IZUMO1 across species, it is likely that bIZUMO1 also functions in relation to the fusion of sperm and egg; however, the biochemical function(s) of the various types of C-terminal cytoplasmic domains remain to be defined.

### Presence of IZUMO1 in bovine sperm

**Figure 4:**
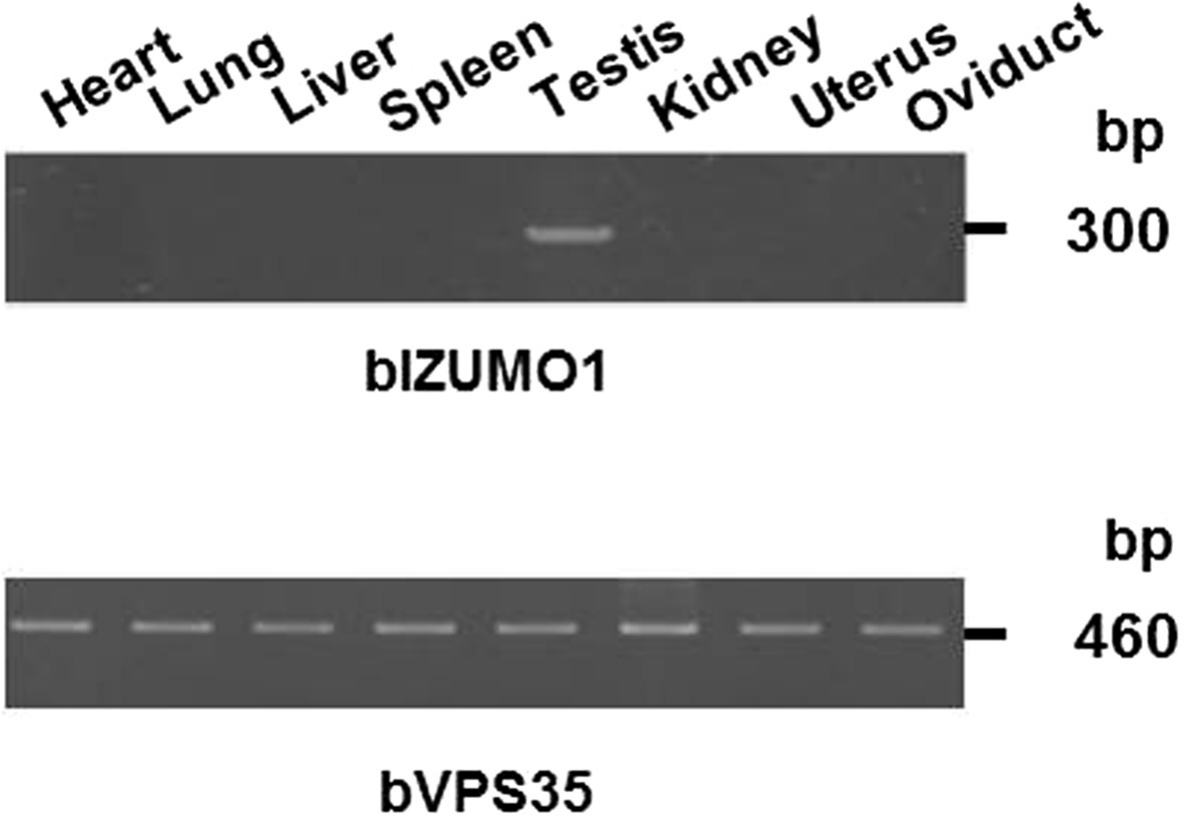
**Expression pattern of IZUMO1 in various bovine tissues obtained by RT-PCR analysis.** Total RNA was isolated from various mouse tissues and subject to RT-PCR analysis with primers specific for bovine VSIG1 and VPS35.

**Figure 5:**
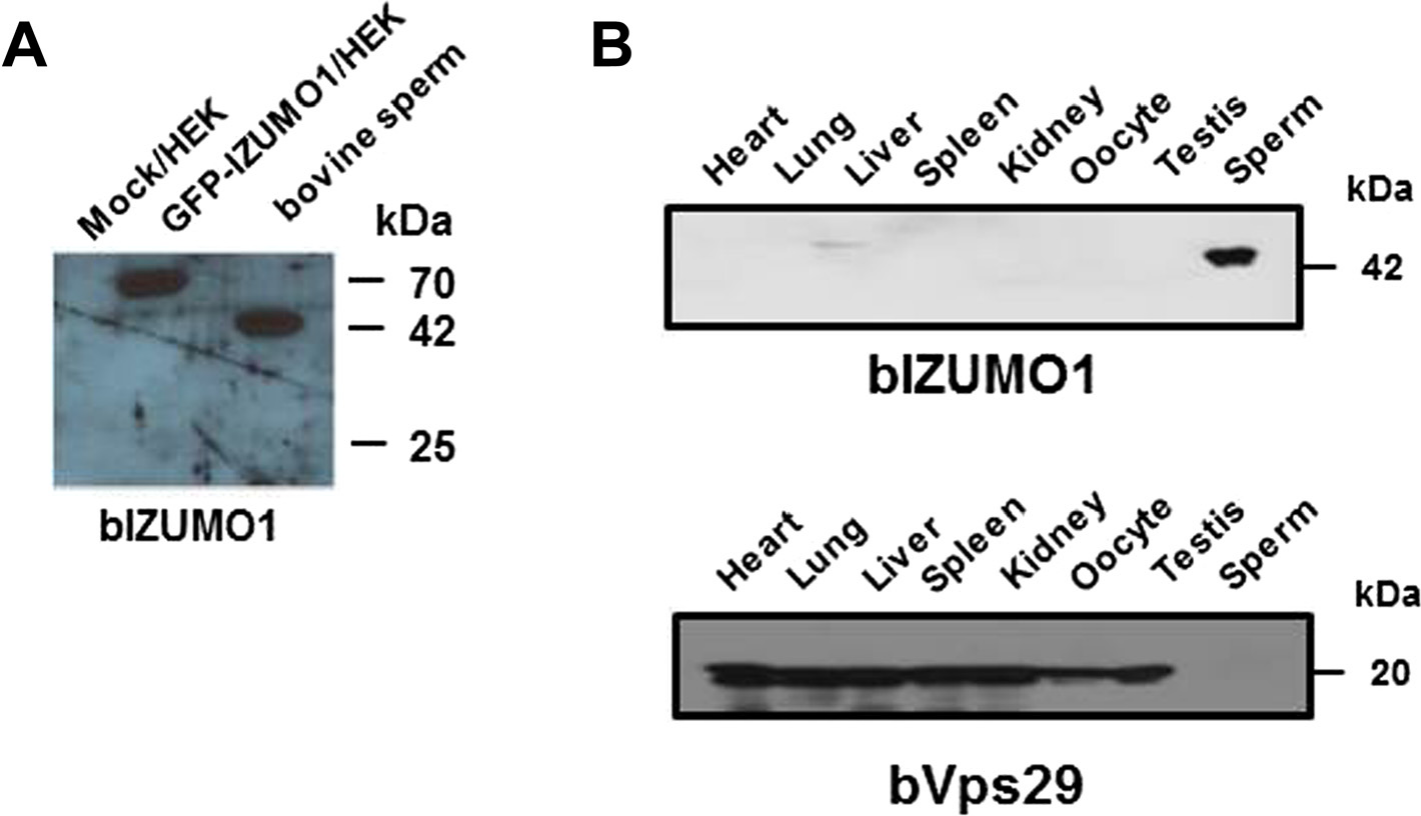
**Localization of IZUMO1 molecule on the bovine sperm. (A)** Protein extracts from HEK293 cells overexpression bIZUMO1 **(A)** and Proteins in Triton X-100 extracts from various bovine tissues **(B)** were separated by SDS-PAGE under reducing conditions and subjected to Western blot analysis using bIZUMO1 and VPS29 antibodies as an internal standard.

Reverse transcriptase-polymerase chain reaction (RT-PCR) analysis indicated that bovine IZUMO1 is expressed specifically in the testis (Figure 4). To examine the expression pattern of bIZUMO1 in bovine tissues, we generated a polyclonal antibody against bovine IZUMO1. The bIZUMO1 antibody immunoreacted with the 42-kDa protein in bovine sperm; no immunoreactive signal for bIZUMO1 was found in the other tissues tested (Figure 5B). For further analysis, we cloned bIZUMO1 into a pEGFP vector and then overexpressed the gene in HEK293 cells [13]. Western blot analysis showed that the anti-bIZUMO1 antibody detected a 65-kDa protein in HEK293 cells transfected with bIZUMO1 (Figure 5A).

### Subcellular localization of IZUMO1 in ejaculated bovine sperm

**Figure 6:**
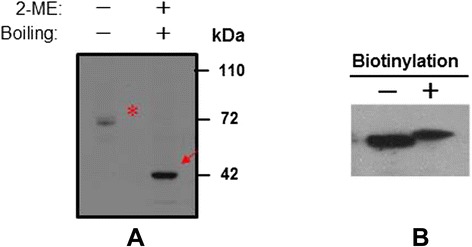
**Dimeric formation of bovine IZUMO1. (A)** Protein extracts from bovine sperm, were separated by SDS-PAGE under non-reducing conditions and subjected to Western blot analysis using an antibody raised against bIZUMO1. Arrows and asterisks indicate the bIZUMO1 complex and monomeric forms, respectively. **(B)** Biotinylation assay of bovine sperm surface molecles: (+) biotin-labeled sperm extracts and (−) non-botinylated sperm extracts.

To date, many sperm membrane proteins have been reported to mediate membrane fusion of the sperm with the egg plasma membrane during fertilization. On the basis of the structure of the bIZUMO1, we predicted that bIZUMO1 belongs to the IgSF family, and thus, it might form complexes on the cell surface. If this were the case, it would verify that IZUMO1 belongs to the IgSF family owing to its structural as well as functional aspects (Figures 1 and 3). To determine whether bIZUMO1 exists on the cell surface, biotinylation assay was carried out using bovine IZUMO1 antibody. As shown Figure 6B, biotinylation of proteins on the surface of bovine sperm and HEK293 cells resulted in slow migration of bIZUMO1 on SDS-PAGE. Therefore bovine IZUMO1 is likely localized on the cell surface. For further analysis, proteins from bovine sperm was denatured by SDS under mild non-reducing conditions and analyzed by western blotting to examine whether bovine IZUMO1 forms a dimeric complex (Figure 6A). The bIZUMO1 antibody recognized immunoreactive signals corresponding to the 42-kDa (monomeric form) as well as to a 70-kDa protein; the latter corresponds to the size of dimeric bIZUMO1, implying that bIZUMO1 forms complex.

## Conclusion

In summary, two major observations were made in the present study. First, we demonstrated the existence of IZUMO1 in bovine sperm. Interestingly, the C-terminal sequence of IZUMO1 is quite different among other species, suggesting that is may have a distinct function. Second, we showed that bIZUMO1 is expressed on the surface of sperm as a homodimeric complex. Inter-IZUMO1 complex formation was recently described during mouse IZUMO1 immunoprecipitation experiments, and it was suggested that the protein forms a homodimeric complex in mouse sperm [13,14]. Indeed, most immunoglobulin superfamily members have been reported to exert their function in various biological processes, such as germ cell migration, transmigration of leucocytes and viral invasion, through homophilic and heterophilic interactions [15,16]. As shown Figure 6, the results from Western blot analysis under non-reducing conditions suggested that bIZUMO1 was involved in homophilic interactions, but it may also interact with other unidentified sperm membrane proteins. This is relevant because although the physiological role of IZUMO1 in the fusion between the sperm and unfertilized egg is relatively well understood, little is known about how its structure is linked with this function. Our observations suggest that the complex formation of IZUMO1 may be necessary for retaining the conformation of the protein, which in turn, might be contributing to sperm-egg fusion in some way. Future investigations on this aspect can help gain clearer insights into the molecular mechanisms underlying fertilization in species of interest.
